# Interrelations of Graph Distance Measures Based on Topological Indices

**DOI:** 10.1371/journal.pone.0094985

**Published:** 2014-04-23

**Authors:** Matthias Dehmer, Frank Emmert-Streib, Yongtang Shi

**Affiliations:** 1 Department of Computer Science, Universität der Bundeswehr München, Neubiberg, Germany; 2 Division for Bioinformatics and Translational Research, UMIT, Hall in Tyrol, Austria; 3 Computational Biology and Machine Learning Laboratory, Center for Cancer Research and Cell Biology, School of Medicine, Dentistry and Biomedical Sciences, Faculty of Medicine, Health and Life Sciences, Queen's University Belfast, Belfast, United Kingdom; 4 Center for Combinatorics and LPMC-TJKLC, Nankai University, Tianjin, China; 5 College of Computer and Control Engineering, Nankai University, Tianjin, China; University of Maribor, Slovenia

## Abstract

In this paper, we derive interrelations of graph distance measures by means of inequalities. For this investigation we are using graph distance measures based on topological indices that have not been studied in this context. Specifically, we are using the well-known Wiener index, Randić index, eigenvalue-based quantities and graph entropies. In addition to this analysis, we present results from numerical studies exploring various properties of the measures and aspects of their quality. Our results could find application in chemoinformatics and computational biology where the structural investigation of chemical components and gene networks is currently of great interest.

## Introduction

Methods to determine the structural similarity or distance between graphs have been applied in many areas of sciences. For example, in mathematics [1], [2], [3], in biology [4], [5], [6], in chemistry [7], [8] and in chemoinformatics [9]. Other application-oriented areas where graph comparison techniques have been employed can be found in [10], [11], [12]. Note that the terms ‘graph similarity’ or ‘graph distance’ are not unique and strongly depend on the underlying concept. The two main concepts which have been explored extensively are exact and inexact graph matching, see [13], [3]. Exact graph matching [2], [3] relates to match graphs based on isomorphic relations. An important example is the so-called Zelinka distance [3] which requires computing the maximum common subgraphs of two graphs with the same number of vertices. However, it is evident that this technique is computationally demanding as the subgraph graph isomorphism problem is NP-complete [14]. In contrast to this, inexact or approximative techniques for comparing graphs match graphs in an error-tolerant way, see [13]. A highlight of this development has been the well-known graph edit distance (GED) due to Bunke [15]. String-based techniques also fit into the scheme of approximative graph comparison techniques [1], [16]. This approach aims to derive string representations which capture structural information of the underlying networks. By using string alignment techniques, one is able to compute similarity scores of the derived strings instead of matching the graphs by using classical techniques. Concrete examples thereof can be found in [1], [16].

As mentioned, numerous graph similarity and distance measures have been explored. But in fact, there is still a lack of a mathematical framework to explore interrelations of these measures. Suppose let 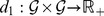 and 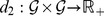 be two comparative graph measures (i.e., graph similarity or distance measures) which are defined on the graph class 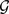. Typical questions in this idea group would be to prove interrelations of the measures by means of inequalities such as 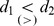. For instance, inequalities involving graph complexity measures have been inferred by Dehmer et al. [17], [18].

The main contribution of this paper is to infer interrelations of graph distance measures. To the best of our knowledge, this problem has not been tackled so far when using graph distance measures. However, interrelations of topological indices interpreted as complexity measures have been studied, see [7], [19], [20], [17], [18]. For instance, Bonchev and his co-workers investigated interrelations of branching measures by means of inequalities [7], [19], [20]. Dehmer [17] examined relations between information-theoretic measures which are based on information functionals and between classical and parametric graph entropies [18]. We here put the emphasis on graph distance measures which are based on so-called topological indices. These measures themselves have not yet been studied. Note that we only consider distance measures (without loss of generality) as they can be easily transformed into graph similarity measures [21]. In order to define these measures concrete, we employ an existing distance measure (see Eq. (6)) and the well-known Randić index [22], the Wiener index [23], eigenvalue-based measures [24], and graph entropies [17], [25]. Also, we discuss quality aspects of the measures and state conjectures evidenced by numerical results.

## Methods and Results

### Topological Indices and Preliminaries

In this section, we introduce the topological indices which are used in the paper. A topological index [23] is a graph invariant, defined by
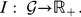
(1)


Simple invariants are for instance the number of vertices, the number of edges, vertex degrees, degree sequences, the matching number, the chromatic number and so forth, see [26].

We emphasize that topological indices are graph invariants which characterize its topology. They have been used for examining quantitative structure-activity relationships (QSARs) extensively in which the biological activity or other properties of molecules are correlated with their chemical structures [27]. Topological graph measures have also been applied in ecology [28], biology [29] and in network physics [30], [31]. Note that various properties of topological graph measures such as their uniqueness and correlation ability have been examined too [32], [33].

Suppose 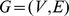 is a connected graph. The distance between the vertices 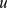 and 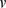 of 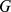 is denoted by 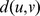. The Wiener index of 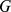 is denoted by 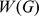 and defined by
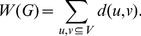
(2)


The name Wiener index or Wiener number for the quantity defined is common in the chemical literature, since Wiener [34] in 1947 seems was the first who considered it. For more results on the Wiener index of trees, we refer to [35].

In 1975, Randić [36] proposed the topological index 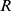 (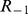 and 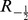) by using the name *branching index* or *connectivity index*, suitable for measuring the extent of branching of the carbon-atom skeleton of saturated hydrocarbons. Nowadays this index is also called the Randić index. In 1998, Bollobás and Erdös [37] generalized this index by replacing 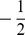 by any real number 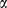, which is called the general Randić index. In fact, the Randić index and the general Randić index became the most popular and most frequently employed structure descriptors used in structural chemistry [38]. For a graph 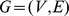, the Randić index 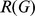 of 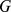 has been defined as the sum of 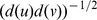 over all edges 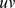 of 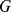, i.e.,
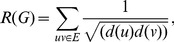
(3)


where 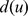 is degree of a vertex 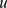 of 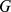. The zeroth-order Randić index due to Kier and Hall [6] is
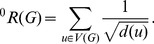
(4)


For more results on the Randić index and the zeroth-order Randić index, we refer to [39], [22], [38].

For a given graph 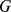 with 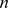 vertices, 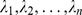 are the eigenvalues of 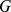. The *energy* of a graph 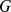, denoted by 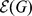, has been defined by
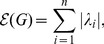
(5)


due to Gutman in 1977 [40]. For more results on the graph energy, we refer to [41], [24], [42].

### Novel Graph Distance Measures

Now we define the distance measure [21]

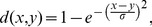
(6)


which is a mapping 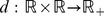. Obviously it holds 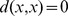, 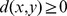, and 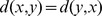. In order to translate this concept to graphs, we employ topological indices and obtain
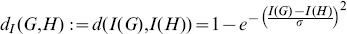
(7)


Further we infer a relation between the maximum value of 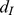 and the extremal values of 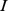.

#### Observation 1

*Let* 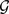 *be a class of graphs.* *Suppose* 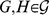, *then* 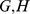 *are the two graphs attaining the maximum value of* 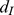 *if and only if* 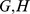 *are the graphs attaining the maximum and minimum value of* 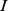, *respectively.*

*Proof*. Let 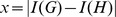, then 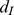 is a monotone increasing function on 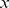. Therefore, the maximum value of 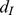 is attained if and only if the maximum value of 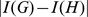 is attained. 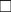


From Observation 1 and some existing extremal results of topological indices, we obtain some sharp upper bounds of 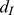 for some classes of graphs. As an example, we list some of those results for trees.

#### Theorem 1

*Let* 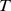 *and* 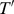 *be two trees with* 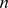 *vertices. Denote by* 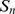 *and* 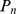 *the star graph and path graph with* 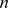 *vertices, respectively.*


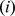
. The maximum value of 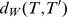 is attained when 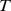 and 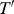 are 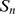 and 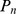, respectively.


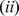
. The maximum value of 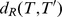 is attained when 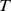 and 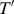 are 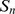 and 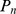, respectively.


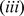
. The maximum value of 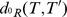 is attained when 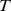 and 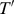 are 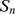 and 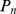, respectively.


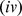
. The maximum value of 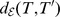 is attained when 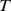 and 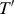 are 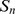 and 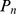, respectively.

### Interrelations of Graph Distance Measures

Observe that 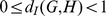, which implies that 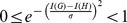. Some trivial properties of 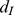 are as follows. Let 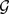 be a class of graphs and 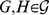. We get
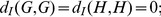
(8)

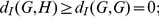
(9)

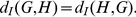
(10)


However, 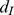 is not a metric graph distance measure, since the triangle inequality 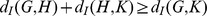 for 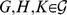, does not hold generally. Actually, we obtain a modified version of the triangle inequality.

#### Theorem 2

*Let* 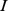 *be a topological index. Let* 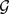 *be a class of graphs and* 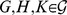. *If*

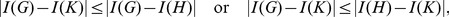
(11)


then we have 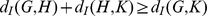.

*Proof.* We now suppose 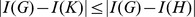, since the proof of the other case is similar.

From the inequality 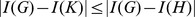, we get
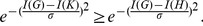
(12)


Since 
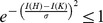
, together with Eq. (12), we have
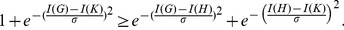
(13)


Therefore, we have the following inequality,
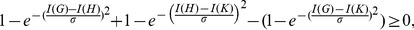
(14)


i.e., 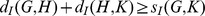. 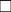


We emphasize if the Inequalities 11 are satisfied, the modified triangle inequality holds. In practice, the triangle inequality may not be absolutely necessary (e.g., for clustering and classification problems) and is often required to prove properties of the measures.

#### Theorem 3

*Let* 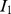 *and* 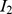 *be two topological indices. Let* 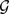 *be a class of graphs and* 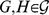. *If*

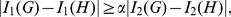
(15)


*then*

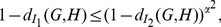
(16)


*where* 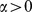 *is a constant.*

*Proof.* Since
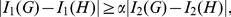
(17)


we obtain
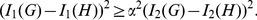
(18)


Thus
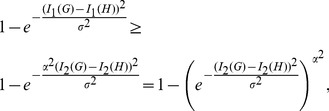
(19)


i.e.,
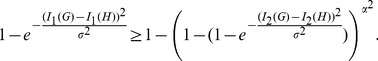
(20)


Thus,
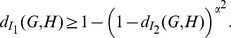
(21)


The proof is complete. 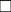


Suppose 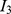 is also a topological index. Then if
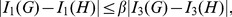
(22)


we derive similarly
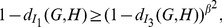
(23)


where 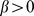 is a constant. Therefore, we obtain the following theorem.

#### Theorem 4

*Let* 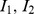 *and* 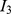 *be three topological indices. Let* 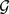 *be a class of graphs and* 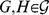. *If*

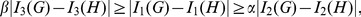
(24)


*then we infer*

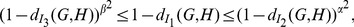
(25)


*where* 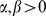 *are constants.*

#### Theorem 5

*Let* 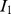 *and* 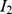 *be two topological indices. Let* 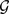 *be a class of graphs and* 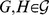. *If*

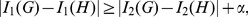
(26)


*then we get*

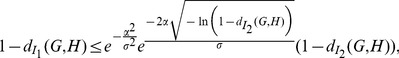
(27)


*where* 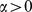 *is a constant.*

*Proof*. Since
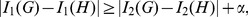
(28)


we infer
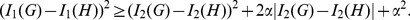
(29)


And therefore,
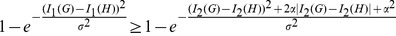
(30)

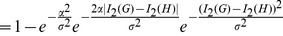
(31)

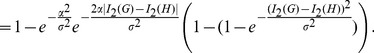
(32)


Hence,
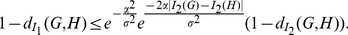
(33)


From the definition of 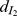, i.e.,
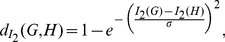
(34)


we obtain that
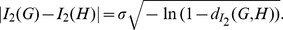
(35)


Finally, by substituting (35) into (33), we get the desired result. 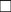


Suppose 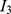 is also a topological index. Then if
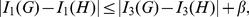
(36)


we have
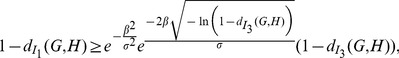
(37)


where 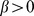 is a constant. Therefore, we obtain the following theorem.

#### Theorem 6

*Let* 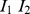 *and* 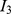 *be three topological indices. Let* 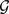 *be a class of graphs and* 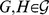. *If*

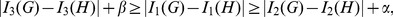
(38)


*then we have*

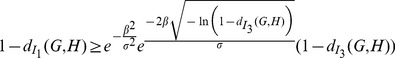
(39)


*and*

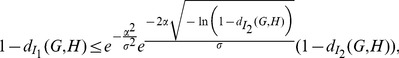
(40)


*where* 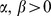 *are constants.*

#### Theorem 7

*Let* 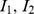 *and* 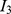 *be three topological indices. Let* 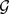 *be a class of graphs and* 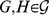. *If*

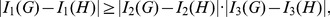
(41)


*then we infer*

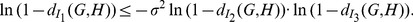
(42)


*Proof.* Since
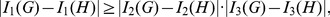
(43)


we derive
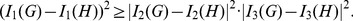
(44)


And therefore,
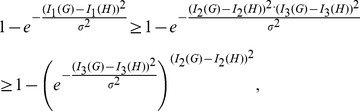
(45)


i.e., 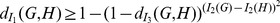. Hence we obtain
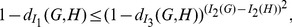
(46)


which implies that
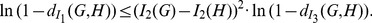
(47)


By substituting (35) into (47), we easily obtain the assertion of the theorem. 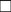


By performing a similar proof as in Theorem 7, we obtain a more general result.

#### Theorem 8

*Let* 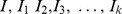 *be topological indices. Let 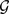 be a class of graphs and* 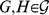. If
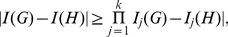
(48)


*we infer*

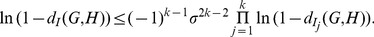
(49)


#### Theorem 9

*Let* 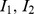 *and* 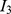 *be three topological indices. Let* 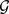 *be a class of graphs and* 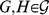. *If*

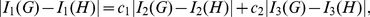
(50)


where 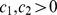, then we get
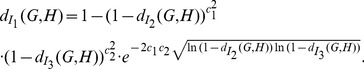
(51)


*Proof.* Since
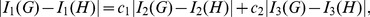
(52)


we derive



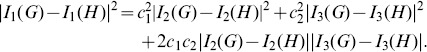
(53)


Therefore,
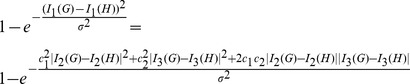
(54)

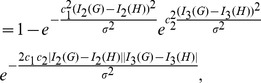
(55)


which implies
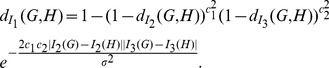
(56)


By applying the substitutions
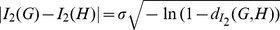
(57)


and
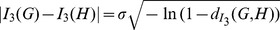
(58)


into (56), we obtain the final result. 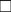


By performing a similar proof as in Theorem 9, we obtain a more general result again.

#### Theorem 10

*Let* 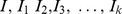 *be topological indices. Let* 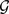 *be a class of graphs and* 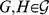. *If*

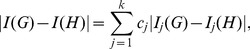
(59)


*where* 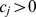 *for* 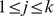, *then we infer*

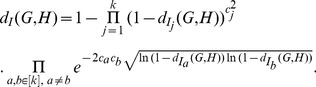
(60)


### Graph Distance Measures Based on Randić Index

In this section, we consider the values of the graph distance measure based on the Randić index and other topological indices for some classes of graphs. Denote by 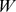 and 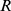 the Wiener index and Randić index, respectively.

#### Theorem 11

*Let* 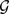 *be a class of regular graphs with* 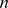 *vertices and* 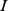 *is an arbitrary topological index. For two graphs* 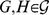, *we infer*

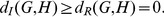
(61)


*Proof.* Let 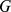 and 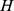 be two regular graphs of order 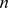. By the definition of the Randić index, we obtain that 
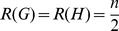
, which implies that 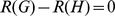. Therefore, we infer 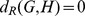. Since 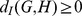 for any topological index, then we obtain the desired inequality.

By using the definition of the zeroth-order Randić index for two graphs with the same degree sequences, we obtain that 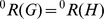. Therefore, we get the following theorem.

#### Theorem 12

*Let* 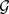 *be a class of graphs with the same degree sequences and* 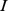 *is an arbitrary topological index. Then for two graphs* 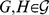, *we infer*

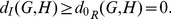
(62)


For a given graph 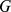 of order 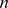, we get 
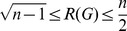
 (see [39]). Thus,
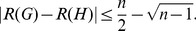
(63)


From (63), we infer an upper bound for 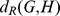.

#### Theorem 13

*Let* 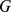 *and* 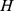 *be two connected graphs of order* 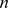. *Then we get*

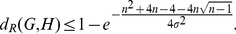
(64)


*The equality holds if and only if* 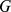 *and* 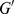 *are* 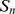 *and a regular graph, respectively.*

A path 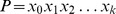 is *pendent* if 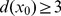, 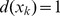 and 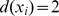 for all 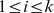. Especially, a vertex 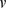 is *pendent* if 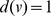. Suppose 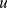 and 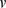 are two pendent vertices, and 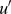 the unique neighbor of 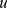. We define an operation as follows: deleting the edge 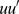 and adding the edge 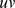. We call this operation “transfer 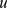 to 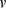
”.

#### Theorem 14

*Let* 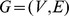 *be a graph with* 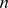 *vertices. Denote by* 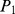 *and* 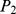 *the two pendent paths attaching to the same vertex such that* 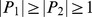. *Denote by* 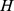 *the graph obtained by transferring the pendent vertex of* 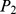 *to the pendent vertex of* 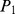. *Then we have*

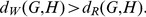
(65)


*Proof.* Let 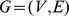 be a graph with 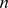 vertices. Suppose 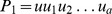 and 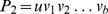 with 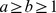. Since 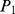 and 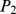 are two pendent paths attaching to the same vertex, then we get
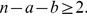
(66)


By using the definition of 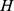, we infer 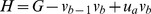. By using the definition of 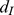, we only need to show
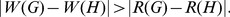
(67)


Observe that 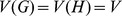. We will discuss the difference of the distances between two vertices in 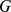 and 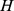. Let 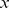 and 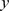 be two vertices of 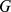. If 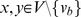, then we have 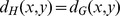. Now we suppose 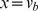. If 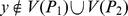, then
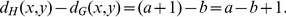
(68)


Observe that
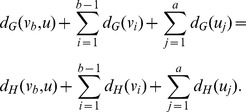
(69)


Therefore, we have
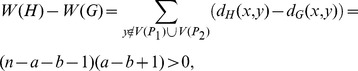
(70)


i.e,
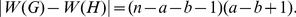
(71)


For 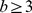, it is easy to verify 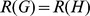. Therefore 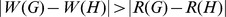 holds.

For 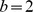, from (66), we have 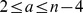 and 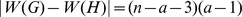. By performing some elementary calculations, we get
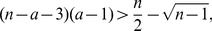
(72)


i.e.,
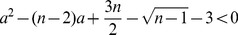
(73)


for 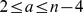 and each value of 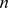. Therefore, from (63), we infer 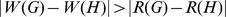.

For 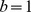, from (66), we have 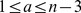 and 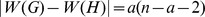. By performing some elementary calculations, we obtain
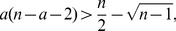
(74)


i.e.,
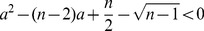
(75)


for 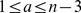 and each value of 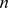. Therefore, from (63), we infer 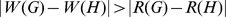. The proof is complete. 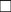


This theorem can be used to compare the values of the distance measure by using trees. Let 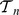 be the set of trees with 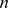 vertices and
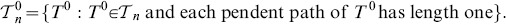
(76)


Observe that for every 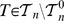, there must be a tree 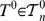 such that 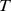 can be obtained from 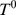 by repeatedly transferring pendent vertices. Therefore, we obtain the following corollary.

#### Corollary 1

*Let* 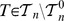, *there exists a tree* 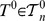 *such that* 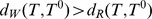.

Actually, numerical experiments show that for any two trees 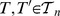, the inequality 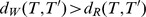 holds. We state the result as a conjecture.

#### Conjecture 1

*Let* 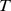 *and* 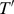 *be any two trees with* 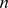 *vertices. Then*

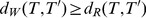
(77)


*holds.*

**Figure 1:**
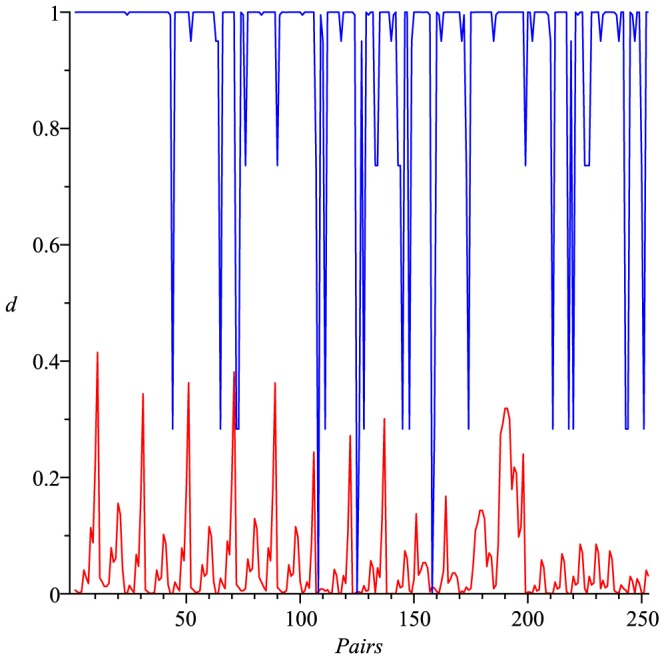
All the values of 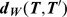 (blue) and 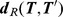 (red). The Y-axis denotes the values of the distance measure and the X-axis denotes the graph pairs.

As an example, we consider (all) 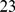 trees with 8 vertices and calculate all possible values of 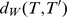 (blue) and 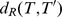 (red) as shown in Figure 1. From Figure 1, we observe that 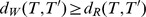 holds for each pair of trees 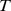 and 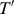.

### Graph Distance Measures Based on Graph Entropy

In this section, we consider graph distance measures which are based on graph entropy and other topological indices for some classes of graphs.

In order to start, we reproduce the definition of Shannon's entropy [43]. Let 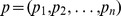 be a probability vector, namely, 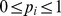 and 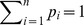. The Shannon's entropy of 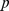 has been defined by
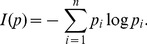
(78)


We denote by 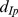 the graph distance measure based on 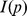.

In the following, we infer an upper bound for 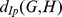.

#### Theorem 15

*Let* 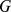 *and* 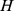 *be two graphs with the same vertex set. Denote by* 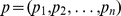 *and* 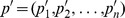 *be the probability vectors of* 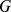 *and* 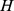, *respectively. If* 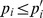 *for each* 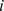, *then we infer*

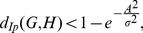
(79)


where 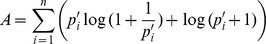.

*Proof.* Since 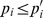 for each 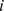, then we obtain 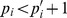 and 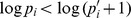. Then we have
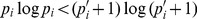
(80)

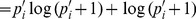
(81)

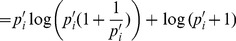
(82)

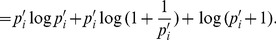
(83)


Therefore, we get the inequality,
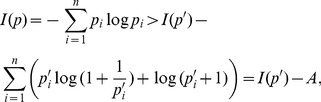
(84)


i.e., 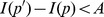. Hence,
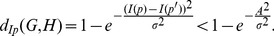
(85)


The desired inequality holds. 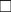


In [25], Dehmer and Mowshowitz generalized the definition of graph entropy by using information functionals. Let 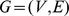 be a connected graph. For a vertex 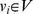, we define
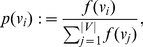
(86)


where 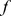 represents an arbitrary information functional. By substituting 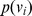 to (78), we have
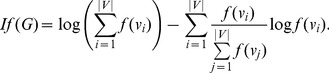
(87)


We denote by 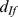 the graph distance measure based on 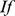.

### Relations between 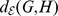 and 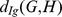


Denote by 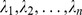 the eigenvalues of a graph 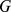. By setting 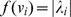 in (87), we obtain a new expression of the graph entropy namely
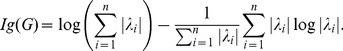
(88)


Recall that the energy of 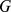 is defined as 
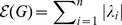
. Then we infer
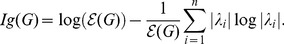
(89)


From the definition of 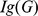, it is interesting to investigate the relation between the graph distance measures 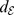 and 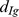.

#### Theorem 16

*Let* 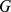 *and* 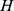 *be two graphs of order* 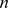 *with* 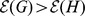. *Denote by* 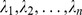 *and* 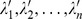 *the eigenvalues of* 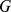 *and* 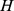, *respectively. Let* 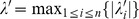 *and* 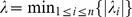. *Then we get*

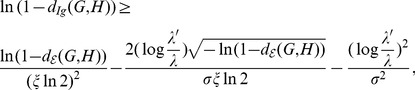
(90)


*where* 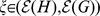 *is a constant.*

*Proof.* Let 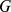 and 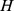 be two graphs of order 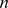. Let 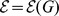 and 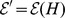 with 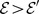. Then we get
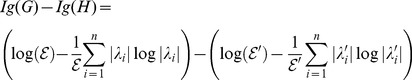
(91)

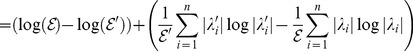
(92)

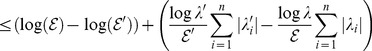
(93)

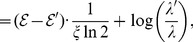
(94)


where 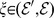. Thus,
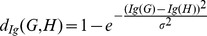
(95)

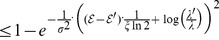
(96)

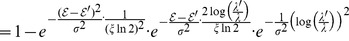
(97)

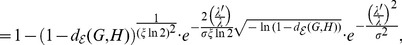
(98)


i.e.,
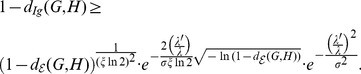
(99)


Taking logarithm for the two sides of the above inequality, we have
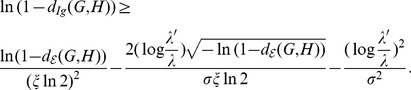
(100)


The required inequality holds. 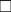


**Figure 2:**
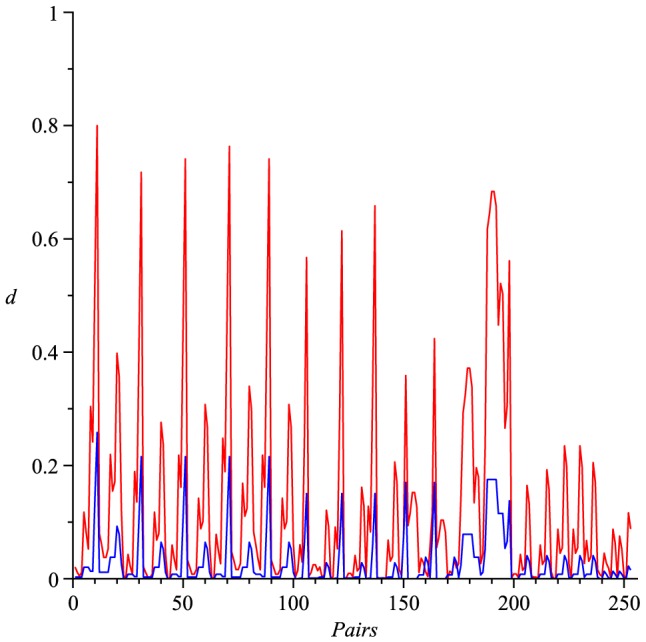
Values of 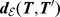 (red) and 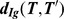 (blue). The Y-axis denotes the values of the distance measure and the X-axis denotes the graph pairs.

Actually, numerical experiments show that for any two distinct trees 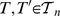, 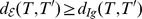 holds. See Figure 2 as an example, in which we consider (all) 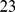 trees with 8 vertices and calculate all possible values of 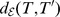 (red) and 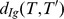 (blue). We state this observation as a conjecture.

#### Conjecture 2

*Let* 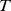 *and* 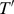 *be any two distinct trees with* 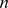 *vertices. Then*

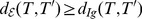
(101)


*holds.*

Using a similar proof method of Theorem 16, we can obtain a generalization for the distance measure based on 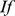 (see Eq. (87)). Let 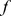 be an arbitrary information functional and 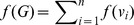 be a topological index.

#### Theorem 17

*Let* 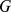 *and* 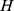 *be two graphs of order* 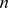 *with* 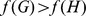. *Let* 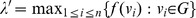 *and* 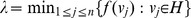. *Then we have*

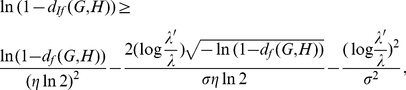
(102)


*where* 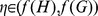 *is a constant.*

Dehmer and Mowshowitz [44] introduced a new class of measures (called here generalized measures) that derive from functions such as those defined by Rényi's entropy and Daròczy's entropy. Let 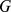 be a graph of order 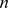. Then
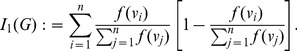
(103)


If we let 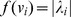, then we can obtain the new generalized entropy based on eigenvalues. We denote the entropy by
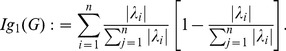
(104)


For a given graph 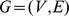 with 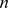 vertices, denote by 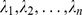 the eigenvalues of 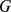. By substituting 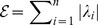 into equality (104), we have
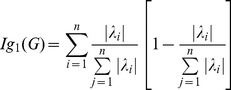
(105)

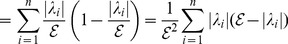
(106)

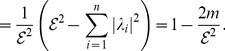
(107)


The last equality holds since 
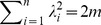
. By the following theorem, we study the relation between 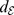 and 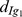.

#### Theorem 18

*Let* 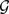 *be a class of graphs with* 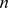 *vertices and* 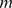 *edges. For two graphs* 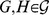, *let* 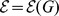 *and* 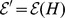. *Then we get*

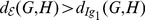
(108)


*and*

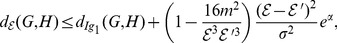
(109)


*where* 
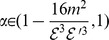
 *is a constant.*

*Proof.* Let 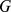 and 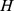 be two graphs with 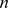 vertices and 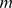 edges. Without loss of generality, we suppose 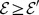.

To show the first inequality, it suffices to prove
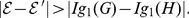
(110)


Then from (107), we derive
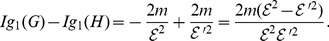
(111)


If we want to prove
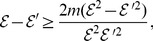
(112)


we only need to show
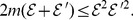
(113)


From a well-known bound of energy 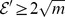, we have 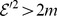 and 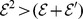. Therefore, 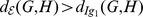 holds.

Now we show the second inequality. From (111), we have
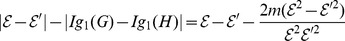
(114)

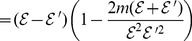
(115)

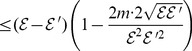
(116)

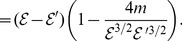
(117)


Therefore, we have
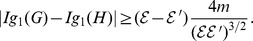



From the definition of the distance measure, by some elementary calculations, we finally infer
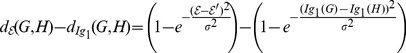
(118)

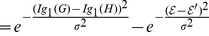
(119)

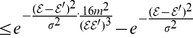
(120)

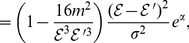
(121)


where 
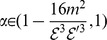
 is a constant.

The proof is complete. 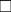


### Relations between 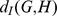 and 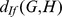


Let 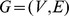 be a connected graph with 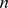 vertices, 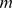 edges and degree sequence 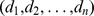, where 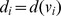 for 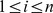. By setting 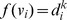 in (87), we can obtain the new entropy based on degree powers, denoted by 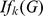


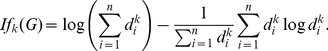
(122)


For 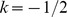, the expression 
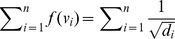
 is just the zeroth-order Randić index 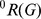. Then by using Theorem 17, we obtain the following result.

#### Theorem 19

*Let* 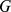 *and* 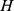 *be two graphs of order* 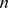 *with* 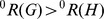. *Let*

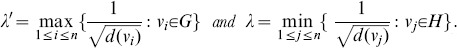
(123)


*Then we have*

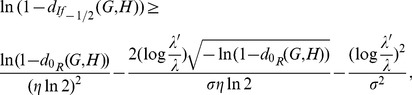
(124)


*where* 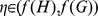 *is a constant.*

For 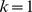, we get
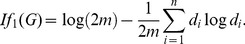
(125)


Furthermore, by the definition of 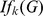, for two graphs with the same degree sequences, we obtain that 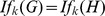. Therefore, we get the following result.

#### Theorem 20

*Let* 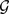 *be a class of graphs with the same degree sequences and* 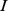 *is an arbitrary topological index. Then for two graphs* 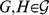, *we infer*

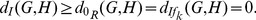
(126)


By using the similar proof method applied in Theorem 14, we obtain a weaker result.

#### Theorem 21

*Let* 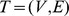 *be a tree with* 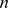 *vertices. Denote by* 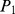 *and* 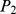 *two pendent paths attaching to the same vertex such that* 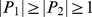. *Denote by* 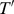 *the tree obtained by transferring the pendent vertex of* 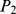 *to the pendent vertex of* 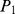. *Then we have*

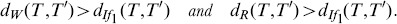
(127)


*Proof.* Let 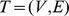 be a tree with 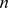 vertices. Suppose 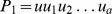 and 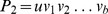 with 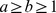. Denote by 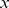 the degree of 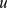, i.e., 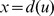. Since 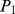 and 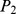 are two pendent paths attaching to the same vertex, then we have 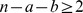. By using the definition of 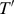, we have 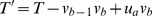. By using the definition of 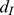, we only need to show
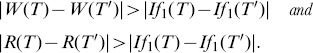
(128)


For a tree 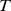 with 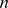 vertices, we get 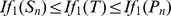. By performing elementary calculations, we get
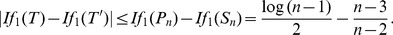
(129)


Observe that 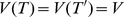. We first discuss the difference of the distances between two vertices in 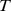 and 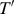. Let 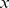 and 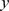 be two vertices of 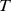. If 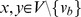, then we have 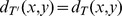. Now we suppose 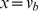. If 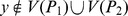, then 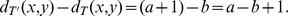 Observe that
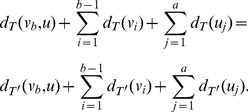
(130)


Therefore, we get 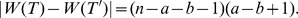


For 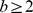, it is easy to verify that 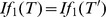, i.e., 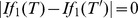. Then,
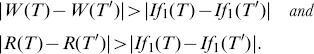
(131)


In the following, we suppose 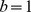.

We obtain 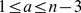 and 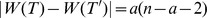. By performing elementary calculations, we get
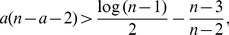
(132)


for 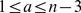 and each value of 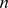. Therefore, 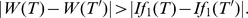


To prove the other inequality, we need more detailed discussion. By using the definition of graph entropy, we get
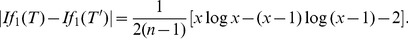
(133)


Let 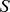 be the set of the neighbors of vertex 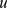, which does not contain 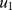 and 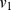. Denote by 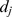 the degree of a vertex in 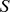, where 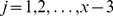. If 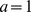, then
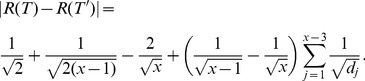
(134)


By performing some calculations, we can show that for 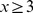 and 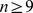,
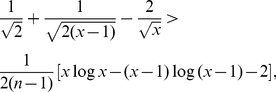
(135)


i.e., 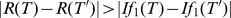 for 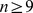. For smaller 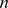, we verify this inequality directly. If 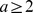, then we have
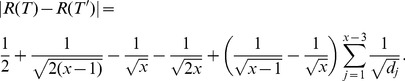
(136)


We can show that for 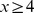 and 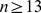,
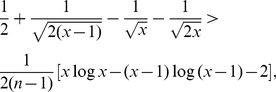
(137)


i.e., 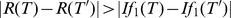 for 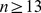. For smaller 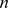, we verify this inequality directly. Now suppose 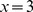, then there is only one vertex in 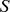 whose degree is at most 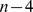. Therefore by using (133) and (136), we get
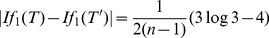
(138)


and
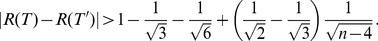
(139)


We can verify
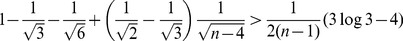
(140)


for each 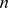, i.e., 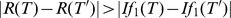. 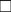


From Theorem 14 and 21, we obtain the following corollary.

#### Corollary 2

*Let* 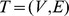 *be a tree with* 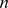 *vertices. Denote by* 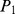 *and* 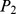 *the two pendent paths attaching to the same vertex such that* 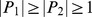. *Denote by* 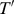 *the tree obtained by transferring the pendent vertex of* 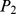 *to the pendent vertex of* 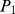. *Then we have*

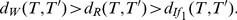
(141)


Therefore, we obtain a similar result to comparing the values of distance measures of trees.

#### Corollary 3

*Let* 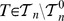, *there exists a tree* 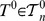 *such that* 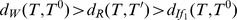.

Actually, our numerical results (see section ‘Numerical Results’) show that for any two trees 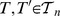, the following inequality may hold.

#### Conjecture 3

*Let* 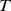 *and* 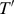 *be any two trees with* 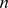 *vertices. Then*

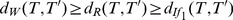
(142)


*holds.*

**Figure 3:**
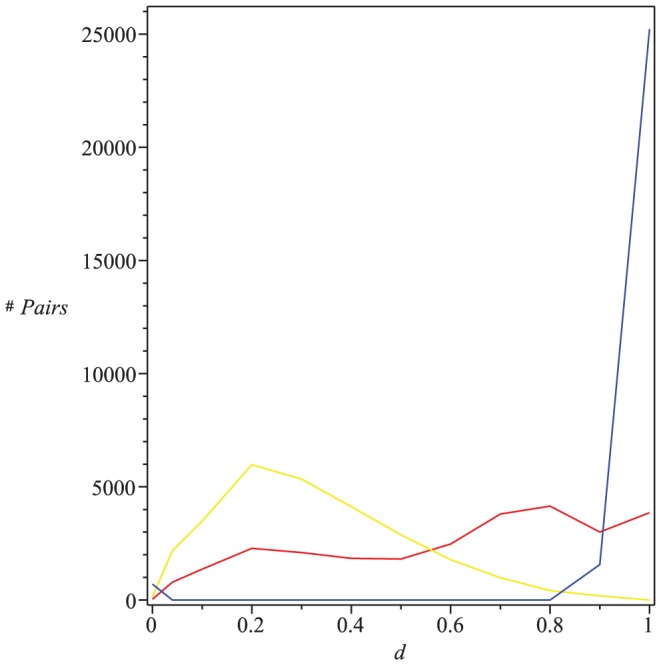
Values of 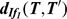 (blue) and 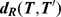 (red). The Y-axis denotes the values of the distance measure and the X-axis denotes the graph pairs.



By way of example, we consider all 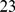 trees of 8 vertices and calculate all possible values of 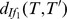 (blue) and 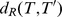 (red), respectively, as shown in Figure 3. From Figure 3, we observe that

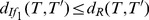
(143)holds for each pair of trees 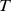 and 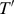.

## Numerical Results

In this section, we interpret the numerical results. First, we consider all trees with 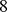 vertices. The number of trees is 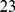 and the number of pairs is 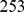 (see [45]). From the curves shown by Figure 1, we see that both measures 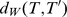 (blue) and 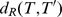 (red) satisfy the inequality Eq. (77). From the curves shown by Figure 2, we observe that both measures 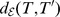 (red) and 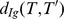 (blue) satisfy the inequality Eq. (101). From the curves shown by Figure 3, we also learn that both measures 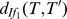 (blue) and 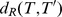 (red) fulfill the inequality Eq. (143). By using this method, several other inequalities could be generated and verified graphically.

**Figure 4:**
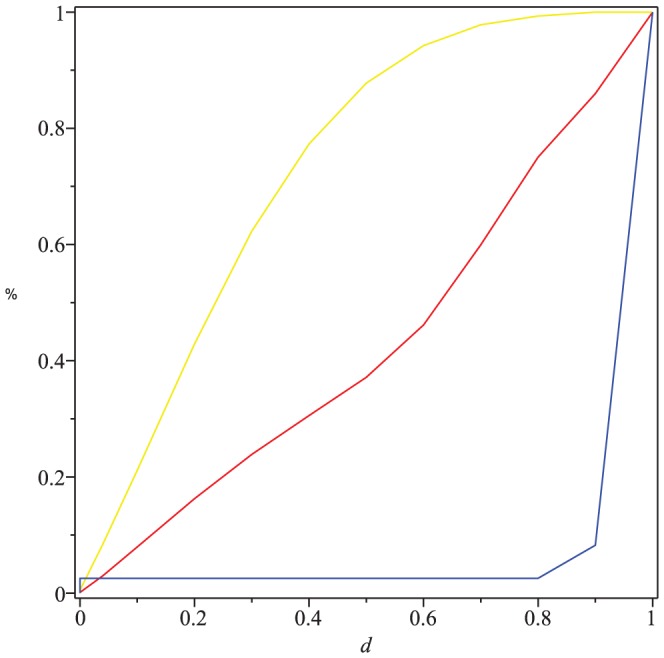
Distributions of the ranked values of the distance measure 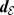 (red), 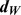 (blue), 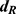 (yellow). The X-axis denotes the values of the distance measure. The Y-axis denotes the number of graph pairs.

**Figure 5:**
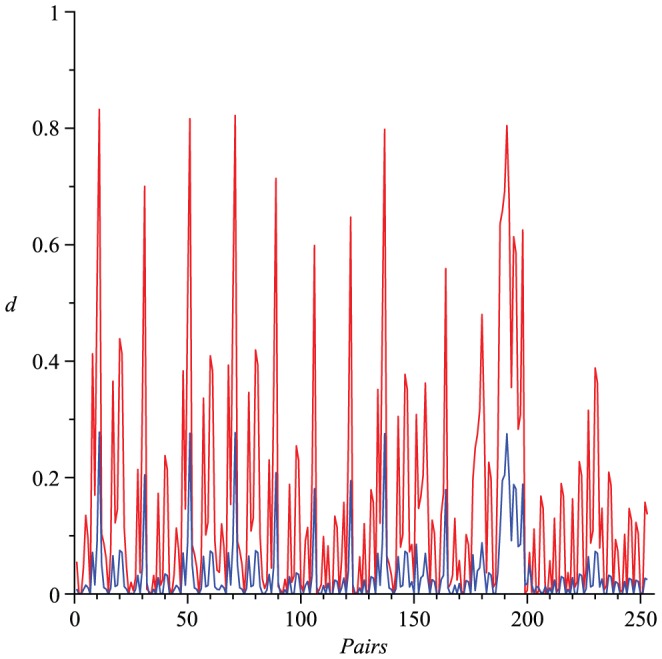
The X-axis denotes the values of the distance measures 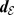 (red), 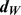 (blue), 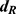 (yellow). The Y-axis represents the percentage rate of all graphs studied.

Figures 4 and 5 show the numerical results by using the graph distance measures based on graph energy 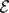, the Wiener index 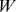 and the Randić index 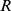, respectively. We consider all trees with 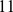 vertices. The number of trees is 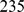 and the number of pairs is 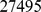 (see [45]). By Figure 4, we depict the distributions of the ranked distance values, that is, 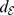 (red), 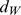 (blue), and 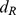 (yellow). First and foremost, we see that the measured values of all three measures cover the entire interval 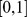. This indicates that the measures are generally useful as they are well defined. By considering 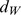, we observe that only a relatively little number of pairs have a measured value 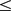 0.8. But a large number of pairs possess distance values 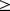 0.8. When considering 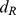, the situation is reverse. The distance values of 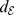 seem to slightly increase with some up- and downturns. However, Figure 4 does not comment on the ability of the graph distance measures to classify graphs efficiently. This needs to be examined in the future and would far beyond the scope of this paper.

Furthermore, we have computed the cumulative distributions by using the measures 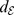 (red), 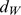 (blue), 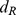 (yellow), respectively, as shown in Figure 5. In general, the computation of the cumulative distribution may serve as a preprocessing step when analyzing graphs structurally. In fact, we see how many percent of the 235 graphs have a distance value which is less or equal 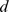. Also, Figure 5 shows that the value distributions are quite different. From Figure 5, we see that the curve for 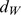 strongly differs from 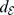 and 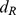. When considering 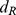, we also observe that about 80% of the 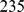 trees have a distance value approximately 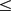 0.5. That means most of the trees are quite dissimilar according to 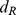. For 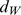, the situation is absolutely reverse. Here 80% of the trees have a distance value approximately 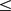 0.98. Finally evaluating the graph distance measure 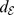 on these trees reveals that about 80% of the trees possess a distance value approximately 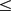 0.85. In summary, we conclude from Figure 5 that all three measures capture the distance between the graphs quite differently. But nevertheless, this does not imply that the quality of one measure may be worse than another. Again, an important issue of quality is fulfilled as the measures turned out to be well defined, see Figure 4. Another crucial issue would be evaluating the classification ability which is future work.

## Summary and Conclusion

In this paper, we have studied interrelations of graph distance measures which are based on distinct topological indices. In order to do so, we employed the Wiener index, the Randić index, the zeroth-order Randić index, the graph energy, and certain graph entropies [25]. In particular, we have obtained inequalities involving the novel graph distance measures. Evidenced by a numerical analysis we also found three conjectures dealing with relations between the distance measures on trees.

From Theorem 1, we see that the star graph and the path graph maximize 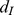 among all trees with a given number of vertices, for any topological index we considered here. Actually, this also holds for some other topological indices, such as the Hosoya index [46], [47], the Merrifield-Simmons index [48], [49], [47], the Estrada index [50], [51], [52], and the Szeged index [53], [54]. All other theorems we have proved in this paper shed light on the problem of proving interrelations of the measures. We believe that such statements help to understand the measures more thoroughly and, finally, they are useful to establish new applications employing quantitative graph theory [55]. We emphasize that the star graph and the path graph are apparently the two most dissimilar trees among all trees. Similar observations can also be obtained for unicyclic graphs or bicyclic graphs. Therefore, in the future, we would like to explore which classes of graphs have this property, i.e., identifying graphs (such as the path graph and the star graph) which maximize or minimize 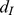.

Another direction for future work is to compare the values of 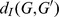 where 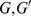 are general graphs. For example, we could assume that 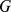 and 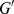 are obtained by only one graph edit operation, i.e., GED(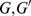) = 1, see [15]. Then, all the graph which fulfill this equation are (by definition) similar. This construction could help to study the sensitivity of the measures thoroughly. Note that similar properties of topological indices have already been investigated, see [56]. As a conclusive remark, we mention that dynamics models on spatial graphs have been studied by Perc and Wang and other researchers, see [57], [58]. It would be interesting to study the distance measures in this mathematical framework as well.

## Supporting Information

Supporting Information S1 **CSV file containing descriptor values of 235 trees by using the Randić index.**
(CSV)

Supporting Information S2 **CSV file containing descriptor values of 235 trees by using graph energy.**
(CSV)

Supporting Information S3 **CSV file containing descriptor values of 235 trees by using the Wiener index.**
(CSV)
